# Interpreting PPV and NPV of Diagnostic Tests with Uncertain Prevalence

**DOI:** 10.5041/RMMJ.10527

**Published:** 2024-07-30

**Authors:** Yakov Ben-Haim, Clifford C. Dacso

**Affiliations:** 1 Faculty of Mechanical Engineering, Technion–Israel Institute of Technology, Haifa, Israel; 2 Baylor College of Medicine, Houston, Texas, USA; 3 Knox Clinic, Rockland, Maine, USA

**Keywords:** Diagnostic tests, NPV, PPV, uncertainty

## Abstract

**Objective:**

Medical decision-making is often uncertain. The positive predictive value (PPV) and negative predictive value (NPV) are conditional probabilities characterizing diagnostic tests and assessing diagnostic interventions in clinical medicine and epidemiology. The PPV is the probability that a patient has a specified disease, given a positive test result for that disease. The NPV is the probability that a patient does not have the disease, given a negative test result for that disease. Both values depend on disease incidence or prevalence, which may be highly uncertain for unfamiliar diseases, epidemics, etc. Probability distributions for this uncertainty are usually unavailable. We develop a non-probabilistic method for interpreting PPV and NPV with uncertain prevalence.

**Methods:**

Uncertainty in PPV and NPV is managed with the non-probabilistic concept of robustness in info-gap theory. Robustness of PPV or NPV estimates is the greatest uncertainty (in prevalence) at which the estimate’s error is acceptable.

**Results:**

Four properties are demonstrated. *Zeroing:* best estimates of PPV or NPV have no robustness to uncertain prevalence; best estimates are unreliable for interpreting diagnostic tests. *Trade-off:* robustness increases as error increases; this trade-off identifies robustly reliable error in PPV or NPV. *Preference reversal:* sometimes sub-optimal PPV or NPV estimates are more robust to uncertain incidence or prevalence than optimal estimates, motivating reversal of preference from the putative optimum to the sub-optimal estimate. *Trade-off between specificity and robustness to uncertainty*: the robustness increases as test-specificity decreases. These four properties underlie the interpretation of PPV and NPV.

**Conclusions:**

The PPV and NPV assess diagnostic tests, but are sensitive to lack of knowledge that generates non-probabilistic uncertain prevalence and must be supplemented with robustness analysis. When uncertainties abound, as with unfamiliar diseases, assessing robustness is critical to avoiding erroneous decisions.

## PLAIN LANGUAGE SUMMARY

Clinical decision-making is a complex interplay of determinants that balances physicians’ knowledge of pathophysiology, the clinical situation, and the effectiveness of data adduced to support the decision. Among the commonly available statistical tools, positive and negative predictive values are among the most valuable and commonly available. The positive predictive value (PPV) of a diagnostic test answers the question: what is the probability that a positive test result for a specific disease indicates the presence of that disease? The PPV is a conditional probability: the probability that the person is actually sick with a specific condition given a positive diagnostic test result for that condition; it is the probability of a “true positive.” Conversely, the negative predictive value (NPV) of a test is the conditional probability that the person is not sick with a specified disease, given a negative diagnostic test result for that disease; it is the probability of a “true negative.” Importantly, the PPV and NPV both depend on an estimate of the prevalence of the disease, which is often quite uncertain, variable from population to population, and a prominent confounder in new or emerging diseases and early in pandemics.

Clinical and public health decision-makers face a fundamental challenge when using PPV or NPV to assess test results. They must make consequential decisions and recommendations, rarely having the depth or breadth of evidence that they desire. They depend on optimal NPV or PPV estimates, either directly or implicitly, in trying to optimize the quality of the decision or recommendation. Benefit can come in the form of a felicitous outcome of diagnosis leading to treatment or even preservation of lives by public health intervention in the face of an emerging pandemic. This benefit is what decision-makers want when making a decision that they consider optimal. But, sadly, the benefit of such a strategy is illusory because of the uncertainty in the underlying prevalence estimates of PPV and NPV. When decision-makers employ optimal estimates of PPV and NPV, they ignore this uncertainty.

Optimizing PPV and NPV estimates ignores uncertainty in the prevalence. A better strategy is less sensitively dependent on optimization and is robust against this uncertainty. In this strategy, the outcome that emerges may not be the best that can be imagined, or even the best that can be practically obtained, but it is one that can be confidently achieved despite uncertainty. Rather than relying on the putatively optimal PPV and NPV estimates, the clinician optimizes the robustness to uncertainty in those estimates, while still achieving a medically meaningful path. This strategy, seeking robustness to uncertainty, is counter-intuitive for the clinician who is trained to provide the “best” to the patient. However, which person with an illness would not trade certainty for an ephemeral and unreliable “best” outcome?

Four major properties characterize robustness to uncertainty rather than relying on the misleading optimization. They are zeroing, trade-off, preference reversal, and antagonism between specificity and robustness.

*Zeroing* asserts that optimized PPV or NPV estimates have no robustness to uncertainty in prevalence since optimal estimates maximally exploit all evidence, whether or not the evidence is correct or erroneous. Asserting that putatively optimal estimates have no robustness to uncertainty is contrary to standard thinking that the best estimates of underlying parameters (for example, prevalence) yield best estimates of functions dependent on those parameters, such as PPV and NPV. The subtle fallacy in this intuition is embodied in the presumption that the best estimate of prevalence will yield the best immunity to error in that estimate. Immunity to error in the estimate—robustness to uncertainty—is distinct from the estimate itself; optimizing one does not optimize the other.

This distinction is demonstrated by the second property: *trade-off* between robustness and error. Robustness to uncertainty increases (which is desirable) as error of the estimate increases (which is undesirable). Thus, robustness curves can be developed that enable specifying the level of PPV or NPV that will not be exceeded despite a significant error in the estimated prevalence. For instance, requiring zero error in PPV has zero robustness to uncertainty in prevalence. However, requiring a PPV error no greater than 0.3 is achieved with robustness to twice the estimated error in prevalence. This trade-off makes the consequences of optimization patently clear to the decision-maker.

If evidence underlying the PPV and NPV estimates is reliable, then optimal estimates of these parameters are also reliable. This is unusual in real-world situations where a subject population is almost certainly different from the population from which the evidence for a test is derived. Examples of this abound in the medical literature (e.g. “normal” hemoglobin values ignore the altitude at which the tested subject lives;^1^ and recent controversies in calculated creatinine clearance emphasize the importance of ethnicity, obscured in a large population^2^). However, if uncertainty is large, then sub-optimal estimates may be preferred over putatively optimal estimates. This is the third property: *preference reversal*. Preference reversal between optimal and sub-optimal estimates arises when sub-optimal estimates are more robust to uncertainty than the putative optima. This demonstrates how the robustness strategy helps the decision-maker. For instance, suppose that the putatively optimal PPV estimate is 0.61. This is the value to use if uncertainty is low or absent. However, this estimate has no robustness to uncertainty (the zeroing property). A specific example shows that, given the prevalence uncertainty, then an estimate of 0.46 for the PPV is substantially robust to this uncertainty. This demonstrates a reversal of preference between the putatively optimal estimate (0.61) and a sub-optimal estimate (0.46) to obtain robustness against uncertain prevalence.

Finally, we demonstrate the fourth property: a *trade-off between specificity and robustness to uncertainty* in prevalence: robustness increases as specificity decreases. This provides an additional tool for the decision-maker.

Although incorporating these four properties into the use of NPV and PPV in medical decision-making appears complex, failure to do so can lead to a loss of confidence in the decision and, at worst, erroneous decisions. Herein we demonstrate how the consideration of robustness to uncertainty can be explicitly incorporated into medical use of PPV and NPV estimates when data are sparse or uncertain. The decision-maker enters the process armed with a new tool allowing the explicit recognition of uncertainty. That uncertainty becomes less obscure and more manageable.

## INTRODUCTION

Regardless of whether an individual is affected as in patient-focused medical care or a community as in public health, decision-making is characterized by severe uncertainty. The decision-maker always desires more information than is available. The SARS-CoV-2 pandemic highlighted this conundrum with uncertainties in diagnosis, clinical course, and treatment, confounding medical decision-makers and resulting in incomplete and often contradictory pronouncements by public health authorities. Simple questions as to whether masks were effective in interrupting viral transmission provoked incorrect and misleading advice with ramifications throughout the population leading to a distrust of health authorities.^3^

Health professionals need to make decisions with available evidence, even when it is incomplete and perhaps faulty. During the pandemic, a key metric was the incidence of disease, yet early efforts at mass testing in the United States failed due to a faulty reagent and delay in certifying clinical laboratories.^4^

In order for a test to be useful in decision-making during an emerging epidemic both the positive and negative predictive value of the test need to be known. But in the onset of an epidemic of a novel pathogen such as SARS-CoV-2, the incidence of the disease will be highly uncertain. This uncertainty results from variability of penetration of the pathogen into the community and variability among communities. Additionally, the false positive and false negative features of a diagnostic test may be only estimated. Finally, the penetration of the test into the community may be variable as a function of social determinants of health, particularly early in the epidemic.^5^

The behavior of the candidate virus strain contributes another uncertainty. In many respiratory viruses, SARS-CoV-2 included, substantial transmission originates in asymptomatic infected individuals. Other confounding variables include viral load, influenced by previous exposure or immunization, where test sensitivity may be reduced.^6^,^7^

These uncertainties result from a lack of knowledge of which probability distributions are unavailable. Yet decisions have to be made. Despite the presence of severe uncertainty, the public’s health is served by cautious but resolute decision-making that can be backed up by invoking the evidence with as much strength as the evidence allows. To that end, both positive and negative predictive values must be considered in light of the non-probabilistic uncertainty of available evidence. The non-probabilistic concept of info-gap robustness will provide a method for interpreting both the positive predictive value (PPV) and the negative predictive value (NPV).

The PPV of a diagnostic test answers the question: what is the probability that a positive test result for a specific disease indicates the presence of that disease in the tested individual? The PPV is a conditional probability: the probability that a person is sick with a specific disease given that the person’s test for that disease is positive; it is the probability of a true positive. The NPV of a test is the conditional probability that the person is not sick with a specified disease, given a negative diagnostic test result for that disease;^8^ it is the probability of a true negative. The PPV and NPV are not themselves diagnostic tests; rather, they characterize diagnostic tests. Taken together, PPV and NPV assess “the probability that the test will give the correct diagnosis.”^9^

If a test’s PPV is high, then clinical intervention may be indicated for people whose results from that test are positive. Similarly, if a test’s NPV is high, then intervention may be counter-indicated for people with negative results from that test. However, both PPV and NPV depend on the incidence or prevalence of the disease, which may be quite uncertain. Hence, the concept of robustness, as developed in info-gap theory, can be used for decision-making under this uncertainty.^10^,^11^

Uncertainty in incidence or prevalence can strongly impact the interpretation of a diagnostic result. For instance, Saraceni et al.^12^ studied fecal occult blood tests for detecting colorectal cancer. Using sensitivity and specificity of 66.7% and 62.3%, respectively, and a prevalence of 10% (Saraceni et al.), one calculates that the PPV was 16%, indicating that a positive test result signaled a 16% chance that cancer was actually present. This is fairly low, so a positive occult blood test may only lead to watchful waiting. However, if the prevalence was 20%, then the PPV would be 31% and colonoscopy may be indicated. Looking at a positive test, we find that moderately small variations in prevalence can have substantial clinical implications of a positive test. Analogous clinical reversals occur for NPV with a prevalence of 90% versus 80%.

Zou^13^ provided a method for calculating the statistical confidence interval (CI) for PPV and NPV and demonstrated CI utility. Info-gap robustness is analogous to, but different from, a statistical CI. Info-gap robustness is the greatest range of uncertainty within which a decision (e.g. a PPV or NPV value) is acceptable or yields the same clinical implication. Info-gap robustness is non-probabilistic and independent of knowledge of probability distributions or other probabilistic assumptions (e.g. statistical independence). Info-gap robustness is relevant when probabilistic models are lacking. Also, info-gap robustness is evaluated for a particular choice of the PPV or NPV value and thus can be used to select between alternative choices of that value.

Why is incidence or prevalence uncertain? Sensitivity and specificity of a diagnostic test are known reasonably well, though Manski^14^ noted some uncertainty associated with these values. In contrast, incidence or prevalence may be highly uncertain. Covid-19 incidence could range from 0.017 to 0.618. Statistical uncertainty in incidence or prevalence may derive from limited sampling, or from false negatives or false positives (if sensitivity or specificity, respectively, is less than unity). Estimated incidence or prevalence is uncertain for unfamiliar diseases or evolving epidemics with uncertain dynamics, a complex pathology unique to the individual, or mutating viral diseases, non-random sampling, heterogeneous population, non-stationarity, and general ignorance about the extent of infection. Finally, incidence or prevalence is usually estimated by diagnostic testing. This is uncertain because the diagnostic test is usually not identical to the disease, but only indicative. These situations derive from a lack of knowledge and generate non-probabilistic uncertainty for which info-gap robustness provides a response.

The following sections will mathematically define PPV and NPV, explore the info-gap robustness to uncertain incidence or prevalence, and then provide conclusions regarding the analysis.

## DEFINITIONS

The *prevalence* is the probability that the disease is present in an individual chosen randomly from the population. Its value, *π*, is highly uncertain, but the best estimate is *π~*.

The *sensitivity* of the test, denoted *σ*, is the conditional probability of a positive test result for a person, given true presence of the disease in that person. Thus, *σ* is a property of the test, and its value is reliably known.

The *specificity* of the test, denoted *ψ*, is the conditional probability of a negative test result for a person, given true absence of the disease in that person. Thus, *ψ* is a property of the test, and its value is reliably known.

The *positive predictive value* (PPV) of the test is the conditional probability that the disease is actually present in a tested person, given a positive test result for that person.

The *negative predictive value* (NPV) of the test is the conditional probability that the disease is actually not present in a tested person, given a negative test result for that person.

From Bayes’ law and the definition of complete probability we can relate PPV to prevalence, sensitivity, and specificity:


Eq. (1) $$\text{PPV}=\frac{\sigma }{\sigma +(1+\psi )\left(\frac{1}{\pi }-1\right)}$$

Likewise, NPV is related to prevalence, sensitivity, and specificity:


Eq. (2) $$\text{NPV}=\frac{\psi }{\psi +(1-\sigma )\frac{\pi }{1-\pi }}$$

Uncertainty in prevalence, *π*, causes uncertainty in PPV and NPV. We assess robustness to that uncertainty and draw operational conclusions.

## PPV ROBUSTNESS TO UNCERTAINTY IN THE PREVALENCE

### Formulation of Info-Gap Uncertainty and Robustness

The best estimate of *π* is *π~*, for which an error estimate is *w_s_*, where *w_s_* is a contextual judgment of error, not a maximum possible error. For instance, expert judgment may be: “The prevalence equals about 0.15, but may err by about thirty percent or more.” Thus *π~*=0.15 and *w_s_*=0.005. We stress that *w_s_* is not an upper bound on the error of the estimate, but only a rough calibration of the error. The actual error may be greater or less than *w_s_*.

We quantify this non-probabilistic uncertainty in the prevalence with an info-gap model of uncertainty, *U*(*h*),^9^ defined in the supplementary material. *U*(*h*) is the set of all prevalence values, *π*, whose deviation from the estimate, *π~*, relative to the error estimate *w_s_*, is no greater than the value *h*. However, the value of *h* is unknown. Thus the info-gap model is not a single set of *π* values, but rather an unbounded family of nested sets of *π* values. Each set is specified by the value of *h*, which is a parameter taking any non-negative value. The set *U*(*h*) is a bounded interval of *π* values. The sets *U*(*h*) become more inclusive as *h* increases, giving *h* its meaning as the horizon of uncertainty. Each set is bounded, but the family of all such sets is unbounded. We stress that the info-gap model does not presume knowledge of a worst case. *U*(*h*) is called a fractional-error info-gap model of uncertainty.

The estimated conditional probability of true disease, that is, the estimate of PPV with the estimated prevalence, *π~*, is denoted PPV. This is the putative optimal estimate of PPV.

Let PPV_e_ denote an expert’s estimate of PPV. This could be PPV, or it could be any other value based on the expert’s judgment. We will evaluate the robustness to uncertainty of various choices of PPV_e_, and use the robustness to indicate when PPV should be chosen as PPV_e_ and when a different value is preferable.

We require that the expert’s estimate, PPV_e_, differ from the true (but unknown) conditional probability, PPV, no more than *ɛ*:


Eq. (3) $$\left|{\text{PPV}}_{\text{e}}-\text{PPV}\right|\leq ɛ$$

That is, *ɛ* is the greatest acceptable error in the expert’s estimate of the conditional probability of disease given a positive result in the diagnostic test. Recall that the unknown true value of PPV depends on the uncertain prevalence, as stated in Eq. (1).

The PPV robustness to this uncertainty is the greatest horizon of uncertainty, *h*, up to which all realizations of the true prevalence, *π*, cause the estimate, PPV_e_, to err no more than *ɛ*. The robustness is defined mathematically in the supplementary material.

The robustness depends on the expert judgment of the PPV, the value PPV_e_. The robustness, however, does *not* depend on the true value of the PPV. As we will see, we can evaluate the robustness to uncertainty without ever knowing the correct PPV. A large value of robustness implies that the corresponding value of PPV_e_ is acceptably accurate over a wide range of uncertainty in prevalence. Conversely, a small value of robustness implies that this value of PPV_e_ is not reliable for interpreting the test. The PPV robustness is derived in section A of the supplementary material.

### Numerical Example

We now discuss PPV robustness curves with the symmetric fractional-error info-gap model. We will explain the concepts of zeroing, trade-off of robustness versus error, and preference reversal among PPV estimates. We will also explore the trade-off between sensitivity and specificity, and between specificity and robustness.

In our numerical example, we assume that sensitivity, *σ*, and specificity, *ψ*, each equal to 0.9. The estimated prevalence is *π~* =0.15 with uncertainty weight *w_s_*=0.05. The putative optimal estimate of PPV is PPV=0.61. We consider three alternative expert judgments, PPV_e_, equal to 0.46, 0.61, and 0.77, where the lower and upper values are about 25% less and 25% more than the putative best estimate, which is 0.61.

#### Zeroing

**Figure 1:**
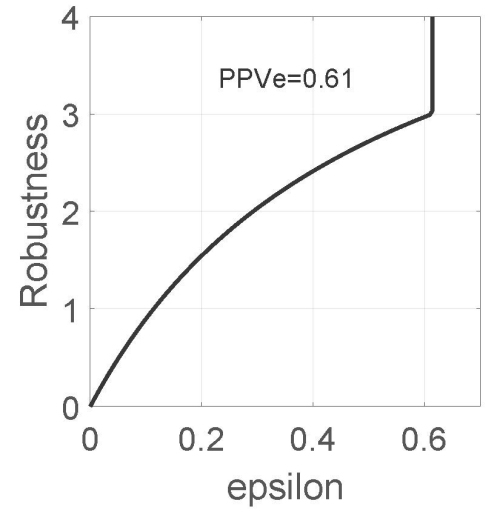
PPV Robustness Function. The sensitivity, *σ*, and the specificity, *ψ*, each take the value 0.9. The estimated prevalence, *π~*, is 0.15, and the estimated error of *π~* is w_s_=*π~*/3. The expert estimate of the positive predictive value, PPV, is denoted PPV_e_. The acceptable error of this estimate is *ɛ*. *σ*=*ψ*=0.9, *π~*=0.15, w_s_=*π~*/3.

The best estimate of the prevalence is *π~*, and the corresponding best estimate of the PPV is based on this estimated prevalence. The zeroing property asserts that predicted outcomes—estimated PPV in the present application—have zero robustness to uncertainty in the data upon which they are based. In Figure 1, the predicted error—the deviation between the true and the best-estimated prevalence—is zero (horizontal intercept); that is, the predicted PPV is presumably reliable. However, the robustness of this predicted error—to uncertainty in the prevalence—is also zero (vertical intercept). A prediction (best PPV estimate) has no robustness to uncertainty in data upon which the prediction depends. This is significant because we usually treat evidence-based predictions as the basis for a decision. However, if the evidence (the value of prevalence in the present situation) is uncertain, then the predicted estimate has no immunity against that uncertainty and would be an unreliable basis for decision-making. We now proceed to identify reliable estimates of the PPV.

#### Trade-off: robustness versus error

The trade-off property asserts that the robustness, *ĥ*_PPV_(s), increases (gets better) as the maximum acceptable error of the estimate is relaxed (*ɛ* increases). This is displayed by the positive slope of the robustness curve in Figure 1. The robustness of the predicted error is zero (the zeroing property), while increasingly positive robustness is obtained by allowing increasingly greater prediction error, *ɛ*.

For example, in Figure 1 we see that if *ĥ*_PPV_ (ɛ) = 1.5 if *ɛ*=0.20. This means that the estimated PPV will err no more than ±0.20 for all values of the prevalence, *π*, in the interval *π~* ± 1.5*w_s_*. This is rather low robustness because the initial judgment was that *π~* could err by ±*w_s_* or more. Hence this estimated PPV is not a reliable basis for decision. Greater robustness is obtained by allowing greater error in the prediction, for any value up to the estimated PPV, which is 0.61. Estimated error equal to or greater than 0.61 is meaningless because it implies that the PPV is between 0 and 1 which is true of any probability value. Up to this estimated error, the robustness trades off against the estimated error: greater error (which is undesirable) has greater robustness to uncertainty (which is desirable).

#### Preference reversal

**Figure 2:**
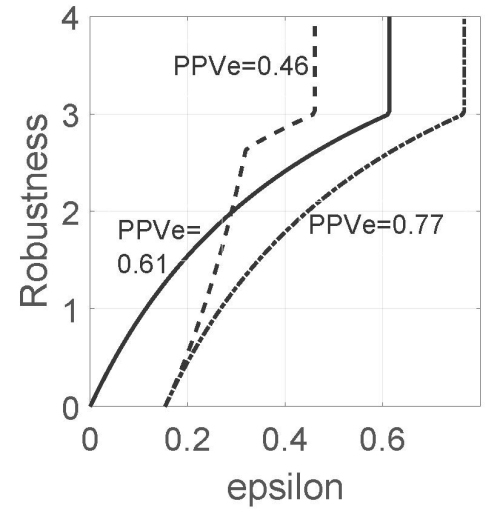
PPV Robustness Functions. The sensitivity, *σ*, and the specificity, *ψ*, each take the value 0.9. The estimated prevalence, *π~*, is 0.15, and the estimated error of *π~* is w_s_=*π~*/3. The expert estimate of the positive predictive value, PPV, is denoted PPV_e_. The acceptable error of this estimate is *ɛ*. *σ*=*ψ*=0.9, *π~*=0.15, w_s_=*π~*/3.

Figure 2 shows robustness curves for three different values of the expert judgment of the conditional probability of disease, PPV_e_: the putative estimate reproduced from Figure 1 (solid curve), and lower and greater values. The robustness curves in Figure 2 show intersection between two of them. We see that the robustness of the sub-optimal estimate PPV_e_=0.46 exceeds the robustness of the putative optimum, PPV=0.61, when *ɛ* exceeds 0.29.

This demonstrates the phenomenon of reversal of preference between options. The putative estimate, PPV=0.61, is based on the available data and is nominally to be preferred for clinical decision-making over any other estimate. However, the estimate PPV=0.46 may be preferred at larger *ɛ* because it is more robust to uncertainty than PPV. Consideration of robustness to uncertainty can result in a reversal of preference from the putative optimum to a sub-optimal but more robust estimate.

#### Trade-off: sensitivity versus specificity

From the expression for PPV in Eq. (1) we see that sensitivity, *σ*, trades off against specificity, *ψ*, at constant PPV. That is, if *σ* increases then *ψ* must decrease to keep the PPV constant, and vice versa. The same trade-off between sensitivity and specificity, at fixed NPV, is seen in Eq. (2). Loh et al.^15^ explore these trade-offs in depth, stressing their importance for selecting a diagnostic test for clinical use. They demonstrate that the numerical strength of the trade-off depends on prevalence of the disease. We consider a variation of this analysis based on consideration of robustness to uncertainty in prevalence.

**Figure 3:**
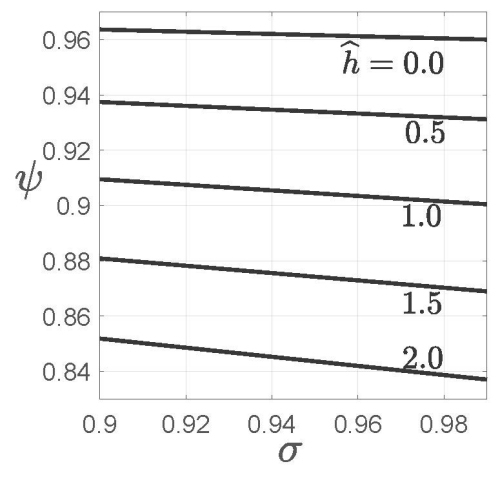
Curves of Constant PPV Robustness. The sensitivity, *σ*, and the specificity, *ψ*. The estimated prevalence, *π~*, is 0.01, and the estimated error of *π~* is w_s_=*π~*/3. The expert estimate of the positive predictive value, PPV, is denoted PPV_e_=0.75 PPV. The acceptable error of this estimate is *ɛ*=0.05. The robustness to uncertainty is denoted *ĥ*. *π~*=0.01, *w_s_*=*π~*/3, *ɛ*=0.05, PPV_e_=0.75 PPV at each (*σ*,*ψ*) point.

Figure 3 shows the trade-off between sensitivity, *σ*, and specificity, *ψ*, when PPV robustness is held constant. The nominal estimate of positive predictive value, PPV, depends on both *σ* and *ψ* and thus varies throughout the (*σ*,*ɛ*) plane. The robustness at each point is evaluated at *PPV_e_*=0.75 PPV. The estimated prevalence is *π~* =0.01, and the acceptable error of the PPV estimate is *ɛ*=0.05. We first explain the construction and meaning of these curves, and then draw an important conclusion.

Each curve in Figure 3 shows that specificity decreases slightly as sensitivity increases relatively more, while robustness is held constant. This derives from the trade-off in the expression for the PPV studied by Loh et al. However, the meaning here is different. In Figure 3 the PPV changes along each curve, unlike in Loh et al., who presented curves of constant PPV. Also, robustness assesses immunity to uncertainty in prevalence, while Loh et al. assumed that prevalence is known. Furthermore, the robustness assesses the immunity to uncertainty in prevalence, for an estimated PPV_e_, which differs from the putative optimum, PPV.

#### Trade-off: specificity versus robustness

The curves in Figure 3 move down as the robustness increases. That is, at fixed sensitivity, *σ*, the specificity, *ψ*, must be *reduced* in order to increase the robustness, with all other parameters at their present values. The test is *more* immune to uncertainty in prevalence at *lower* specificity, for the range of values considered. This may be counter-intuitive at first. However, specificity is the probability of a negative test result from a healthy individual. Here, a very low estimated prevalence is being considered, so most tests are negative. Lowering the specificity will increase the rate of false positives, which simulates the effect of lower prevalence and thus compensates for the uncertain possibility that prevalence is higher than estimated. In this way, lower *ψ* increases the robustness to uncertain prevalence at fixed *σ*, when prevalence is very low and the other parameters are at their present values.

A dilemma is faced when choosing a specificity value if the estimated prevalence is low. On the one hand, high specificity is an inherently desirable attribute of a diagnostic test, partly because the estimated PPV increases as specificity increases, as shown in Eq. (1). On the other hand, robustness to uncertainty in prevalence is also desirable because it enhances confidence in the estimate, but robustness decreases as specificity increases when the estimated prevalence is low. One approach to resolving this dilemma begins by choosing an acceptable error in estimating the PPV, for example *ɛ*=0.05 or 0.10. One then chooses the largest specificity, *ψ*, for which the corresponding robustness to uncertainty, *ĥ*_PPV_(ɛ), is large enough to instill confidence in the PPV.

#### Relative impact on PPV robustness of sensitivity versus specificity

Sensitivity, *σ*, has much lower impact on PPV robustness than specificity, *ψ*, if both are fairly close to unity. This is because the PPV depends far more strongly on *ψ* than on *σ* for relevant parameter values, as seen by the following relation. Using the expression for the PPV in Eq. (1), one finds:


Eq. (4) $$\frac{\partial \text{PPV}/\partial \psi }{\partial \text{PPV}/\partial \sigma }=\frac{\sigma }{1-\psi }$$

For example, this ratio equals 9 if *σ= ψ=* 0.9 and approaches infinity as *ψ* approaches unity for any fixed positive value of *σ*.

## NPV ROBUSTNESS TO UNCERTAINTY IN THE PREVALENCE

An expression for NPV robustness can now be developed, and its implications studied, in analogy to the section “PPV Robustness to Uncertainty in the Prevalence” (page 46). Here again the symmetric fractional-error info-gap model is used.

### Formulation of Info-Gap Robustness

The estimated conditional probability of no disease given a negative test result, that is, the putative best estimate of NPV based on estimated prevalence, *π~*, is, from Eq. (2):


Eq. (5) $$NPV=\frac{\psi }{\psi +(1-\sigma )\frac{\tilde{\pi }}{1-\tilde{\pi }}}$$

Let NPV_e_ denote an expert’s judgment of the value of NPV. This could be NPV or any other value. In analogy to Eq. (3) the performance requirement is:


Eq. (6) $$\left|{\text{NPV}}_{\text{e}}-\text{NPV}\right|\leq ɛ$$

The term *ɛ* is the greatest acceptable error in the expert judgment, NPV_e_, of the conditional probability that the individual is free of disease, given a negative test result. Recall that the unknown true value of NPV depends on uncertain prevalence, as stated in Eq. (2).

The NPV robustness is the greatest horizon of uncertainty, *h*, up to which all realizations of the true prevalence, *π*, cause the judgment, NPV_e_, to err no more than *ɛ*, defined mathematically in the supplementary material. This is the NPV analog of the PPV robustness defined earlier. The NPV robustness is derived in section B of the supplementary material.

### Numerical Example

Now a numerical example of NPV robustness with the fractional-error info-gap model is discussed. Sensitivity and specificity values of *σ*=*ψ*=0.9 are assumed. The estimated value of prevalence is *π~* = 0.15, with an uncertainty weight of *w_s_*=0.05.

**Figure 4:**
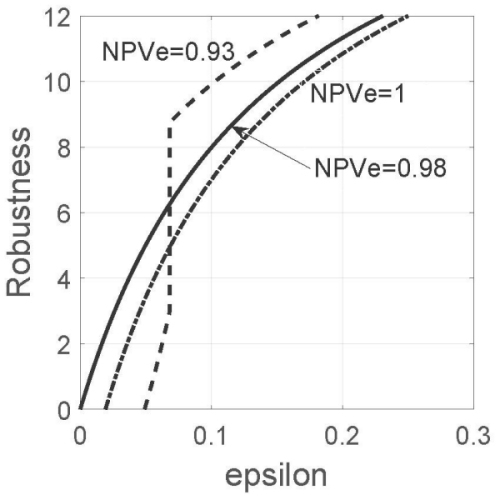
NPV Robustness Functions. The sensitivity, *σ*, and the specificity, *ψ*, each take the value 0.9. The estimated prevalence, *π~*, is 0.15, and the estimated error of *π~* is w_s_=*π~*/3. The expert estimate of the negative predictive value, NPV, is denoted NPV_e_. The acceptable error of this estimate is *ɛ*. *σ*=*ψ*=0.9, *π~*=0.15, w_s_=*π~*/3.

The best estimate of NPV, with these coefficients, is NPV=0.98. This is the estimated probability that a person, whose diagnostic test was negative, is in fact free of disease. This is an encouragingly large value, though we must now consider robustness to uncertainty in prevalence. Figure 4 shows NPV robustness curves for three values of the expert’s judgment of the NPV: NPV_e_=0.93, 0.98 (which is the putative estimate, NPV), and 1.00.

#### Cost of robustness

A distinctive feature of the robustness curves in Figure 4 is their steep slope at low values of *ɛ*. The steep slope implies a low cost of robustness: the robustness increases greatly by increasing the allowed error, *ɛ*, only slightly. For instance, the robustness for the estimated NPV increases from *ĥ*_NPV_(*ɛ*) = 0 to *ĥ*_NPV_(*ɛ*) = 5.0 as *ɛ* increases from 0 to 0.05. A robustness of 5.0 means that the true prevalence can deviate from its estimated value by ±5.0*w_s_* (subject to non-negativity) and the true NPV will deviate from its estimate by no more than 0.05. This NPV value is a reliable basis for decision.

#### Preference reversal

Figure 4 shows that NPV_e_=0.98 is robust dominant over NPV_e_=1 throughout the range of the figure. In contrast, comparison of NPV_e_=0.93 and NPV_e_=0.98 shows a reversal of preference, depending on the allowed error, *ɛ*: NPV_e_=0.98 is more robust than NPV_e_=0.93 at low *ɛ*, and the robustness-based preference is reversed at larger *ɛ*. It is significant that this robust preference for the sub-optimal estimate, NPV_e_=0.93, occurs at fairly low error, *ɛ*. This is different from the preference reversal in the PPV robustness example in Figure 2, which occurs at larger *ɛ*.

Nonetheless, the putative estimate, NPV=0.98, has large robustness at quite small *ɛ* and can therefore provide a reliable basis for evaluating the diagnostic test. This large probability of absence of disease, given a negative test result, would seem to strongly support refraining from medical intervention for a patient whose test result is negative. Moreover, this example demonstrates that the clinician can rely on the putative estimate, NPV. This is unlike the PPV (in this example) where expert judgment, PPV_e_, should perhaps prevail over the putative estimate, PPV.

#### Robustness of NPV and PPV

The PPV is less robust to uncertainty in prevalence than NPV, when the prevalence is low; the situation is reversed when prevalence is high.

#### Relative impact on NPV robustness of sensitivity versus specificity

We saw that sensitivity, *σ*, has much lower impact than specificity, *ψ*, on PPV robustness. The situation is reversed when considering NPV robustness. However, in any case NPV is much less sensitive than PPV to change in either *σ* or *ψ*.

## CONCLUSION

Positive predictive value (PPV) and negative predictive value (NPV) are conditional probabilities that characterize any specific diagnostic test. The PPV is the probability that a tested patient is ill with a specified disease, given a positive test result for that disease; NPV is the probability that a tested patient is not ill with a specified disease, given a negative test result for that disease. Both values depend on disease prevalence, which may be quite uncertain for many reasons. Consequently, PPV and NPV values evaluated with the best estimate of prevalence may err substantially and be an unreliable basis for evaluating the test.

This paper focused on modeling and managing uncertainty in PPV and NPV resulting from uncertain prevalence. Our analysis employed robustness to uncertainty as developed in info-gap theory, motivated by situations when probability distributions are lacking. We demonstrated four properties of interpreting PPV and NPV values resulting from uncertain prevalence, underlying the interpretation of PPV and NPV values.

*Zeroing* asserts that best PPV or NPV estimates lack robustness to uncertainty in prevalence at zero estimation error. This means that best estimates may not be reliable for interpreting positive or negative test results.

This was demonstrated by the *trade-off* between robustness and error: robustness increases (which is desirable) as error increases (which is undesirable). This trade-off was manifested by the positive slopes for the robustness curves in Figures 1, 2, and 4. Robustness curves enable the investigator to identify the level of PPV or NPV error that will not be exceeded despite great error in the estimated prevalence.

The solid and dashed curves in Figure 2 displayed the phenomenon of *preference reversal* between optimal and sub-optimal estimates. At low values of acceptable error, *ɛ*, we see that the putative estimate, PPV=0.61, is more robust and hence preferred over the sub-optimal estimate PPV_e_=0.46. However, if larger *ɛ* is acceptable, or if greater robustness to uncertainty is required, then PPV_e_=0.46 is robust-preferred over PPV=0.61. Neither PPV estimate is robust dominant over the other; the preference between them is reversed as requirements change. This potential for reversal of preference is manifested in the intersection between the robustness curves of the alternative estimates of PPV. In contrast, PPV=0.61 is robust dominant over PPV=0.77; their robustness curves do not cross one another.

Finally, a *trade-off* between specificity and robustness to uncertainty in prevalence was demonstrated: robustness increases as specificity decreases. This may justify using tests with less than maximal specificity, in order to counter the adverse impact of uncertain prevalence.

Taken together, these four properties, employed in the examples herein, can improve individual-patient decision-making as well as community-based policy-making by pointing toward a methodology that provides medically meaningful outcomes robust to uncertainty. While it may not be the best possible outcome conceived or obtainable, it will reliably “get the job done.” Refocusing decision-making on *what can be* rather than on *what might be* is a useful and reliable pathway for both individual patient care and for policy-making.

## Supplementary Information





## Abbreviations

NPV: negative predictive value
PPV: positive predictive value
