# Supplementary material for: Interpreting PPV and NPV of Diagnostic Tests with Uncertain Prevalence
Source: Rambam Maimonides Med J. 2024 Jul 30;15(3):e0013. doi: 10.5041/RMMJ.10527 (PMC11294685; doi:10.5041/RMMJ.10527)
Supplement: Supplementary file 1 [file rmmj-15-3-e0013AM.docx]

This appendix has been provided by the authors for the benefit of readers

Supplement to Interpreting PPV and NPV of Diagnostic Tests with Uncertain Prevalence

Ben-Haim Y, Dacso CC. Interpreting PPV and NPV of Diagnostic Tests with Uncertain Prevalence. Rambam Maimonides Med J 2024;15 (3):. doi:10.5041/RMMJ.

# A. Evaluation of the robustness of the PPV

We employ the following fractional-error info-gap model of uncertainty, discussed in the text:

Supp. Eq. (1)

The PPV robustness of the estimate $\text{PPV}_{\text{e}}$ is defined:

Supp. Eq. (1a)

Let $m(h)$ denote the inner maximum in this definition of the robustness. We note that $m(h)$ is an increasing function of $h$ because the uncertainty sets, *U(h),* become more inclusive as $h$ increases. The robustness is the greatest horizon of uncertainty, $h$, up to which $m(h)$ does not exceed $\varepsilon$. The robustness is less than any value of $h$ for which $m(h)$exceeds $\varepsilon$. Likewise, the robustness exceeds any value of $h$ for which $m(h)$ is less than $\varepsilon$. This means that plotting $h$ versus $m(h)$ is identical to plotting $\hat{h}_{\text{PPV}}(\varepsilon)$ versus $\varepsilon$. In other words, $m(h)$ is the inverse function of the robustness function. Thus it is sufficient to evaluate $m(h)$.

From Supp. Eq. (1) we see that the PPV is monotonic in $\pi$. Hence the inner maximum in the definition of the robustness occurs for an extremal value of the prevalence, $\pi$, either minimal or maximal. Denote the two resulting values of $m(h)$ by $m_{+}(h)$ and $m_{-}(h)$. The value of $m(h)$ is the greater of these two alternatives:

Supp. Eq. (1b)

Note that this maximum may switch between $m_{+}(h)$ and $m_{-}(h)$ as $h$ changes.

Based on Supp. Eq. (1) and the fractional-error info-gap model, we find the following explicit expressions:

Supp. Eq. (1c)

Supp. Eq. (1d)

where we have defined the function *x*^+^=0 if *x*<0, *x*^+^= *x* if 0≤ *x*≤ 1, and *x*^+^=1 else.

# B. Evaluation of the Robustness of the NPV

The NPV robustness of the estimate $\text{NPV}_{\text{e}}$ is defined:

Supp. Eq. (2)

Let $M(h)$ denote the inner maximum in this definition of the robustness. $M(h)$ is the inverse of the NPV robustness function. From Supp. Eq. (2) we see that the NPV is monotonic in $\pi$. Hence, this inner maximum occurs for an extremal value of the prevalence, $\pi$, either minimal or maximal. Denote the two resulting values of $M(h)$ by $M_{+}(h)$ and $M_{-}(h)$. The value of $M(h)$ is the greater of these two alternatives:

Supp. Eq. (2a)

Note that this maximum may switch between $M_{+}(h)$ and $M_{-}(h)$ as $h$ changes.

Based on Supp. Eq. (2) and the fractional-error info-gap model, we have the following explicit expressions:

Supp. Eq. (2b)

Supp. Eq. (2c)

We are again using the function $x^{+}$ defined earlier.
