# Supplementary material for: Interpreting PPV and NPV of Diagnostic Tests with Uncertain Prevalence
Source: Rambam Maimonides Med J. 2024 Jul 30;15(3):e0013. doi: 10.5041/RMMJ.10527 (PMC11294685; doi:10.5041/RMMJ.10527)
Supplement: Supplementary file 2 [file rmmj-15-3-e0013AM.pdf]

SUPPLEMENTARY MATERIAL

This appendix has been provided by the authors for the benefit of readers

Supplement to Interpreting PPV and NPV of Diagnostic Tests with Uncertain Prevalence

Ben-Haim Y, Dacso CC. Interpreting PPV and NPV of Diagnostic Tests with Uncertain Prevalence. Rambam Maimonides Med J 2024;15 (3):e0013. doi:10.5041/RMMJ.10527

A. EVALUATION OF THE ROBUSTNESS OF THE PPV

We employ the following fractional-error info-gap model of uncertainty, discussed in the text:

$$U(h) = \left\{ \pi : \quad \pi \in [0,1], \ \left| \frac{\pi - \tilde{\pi}}{w_s} \right| \le h \right\}, \quad h \ge 0 \qquad \text{Supp. Eq. (1)}$$

The PPV robustness of the estimate PPV_e is defined:

$$\hat{h}_{\text{PPV}}(\varepsilon) = \max \left\{ h : \quad \left(\max_{\pi \in U(h)} \left| \text{PPV}_e - \text{PPV} \right| \right) \le \varepsilon \right\} \qquad \text{Supp. Eq. (1a)}$$

Let $m(h)$ denote the inner maximum in this definition of the robustness. We note that $m(h)$ is an increasing function of h because the uncertainty sets, *U(h),* become more inclusive as h increases. The robustness is the greatest horizon of uncertainty, h, up to which $m(h)$ does not exceed ε. The robustness is less than any value of h for which $m(h)$ exceeds ε. Likewise, the robustness exceeds any value of h for which $m(h)$ is less than ε. This means that plotting h versus $m(h)$ is identical to plotting $\hat{h}_{\text{PPV}}(\varepsilon)$ versus ε. In other words, $m(h)$ is the inverse function of the robustness function. Thus it is sufficient to evaluate $m(h)$.

From Supp. Eq. (1) we see that the PPV is monotonic in π. Hence the inner maximum in the definition of the robustness occurs for an extremal value of the prevalence, π, either minimal or maximal. Denote the two resulting values of $m(h)$ by $m_+(h)$ and $m_-(h)$. The value of $m(h)$ is the greater of these two alternatives:

$$m(h) = \max \left\{ m_-(h), \ m_+(h) \right\} \qquad \text{Supp. Eq. (1b)}$$

Note that this maximum may switch between $m_+(h)$ and $m_-(h)$ as h changes.

Based on Supp. Eq. (1) and the fractional-error info-gap model, we find the following explicit expressions:

$$m_+(h) = \left| \mathrm{PPV_e} - \frac{\sigma}{\sigma + (1-\psi)\left(\dfrac{1}{(\tilde{\pi} + w_s h)^+} - 1 \right)} \right|$$ Supp. Eq. (1c)

$$m_-(h) = \left| \mathrm{PPV_e} - \frac{\sigma}{\sigma + (1-\psi)\left(\dfrac{1}{(\tilde{\pi} - w_s h)^+} - 1 \right)} \right|$$ Supp. Eq. (1d)

where we have defined the function $x^+=0$ if $x<0$, $x^+ = x$ if $0 \le x \le 1$, and $x^+=1$ else.

B. EVALUATION OF THE ROBUSTNESS OF THE NPV

The NPV robustness of the estimate $\mathrm{NPV_e}$ is defined:

$$\hat{h}_{\mathrm{NPV}}(\varepsilon) = \max\left\{ h: \quad \left(\max_{\pi \in U(h)} \left| \mathrm{NPV_e} - \mathrm{NPV} \right| \right) \le \varepsilon \right\}$$ Supp. Eq. (2)

Let $M(h)$ denote the inner maximum in this definition of the robustness. $M(h)$ is the inverse of the NPV robustness function. From Supp. Eq. (2) we see that the NPV is monotonic in π. Hence, this inner maximum occurs for an extremal value of the prevalence, π, either minimal or maximal. Denote the two resulting values of $M(h)$ by $M_+(h)$ and $M_-(h)$. The value of $M(h)$ is the greater of these two alternatives:

$$M(h) = \max\left\{ M_-(h),\ M_+(h) \right\}$$ Supp. Eq. (2a)

Note that this maximum may switch between $M_+(h)$ and $M_-(h)$ as h changes.

Based on Supp. Eq. (2) and the fractional-error info-gap model, we have the following explicit expressions:

$$M_+(h) = \left| \mathrm{NPV_e} - \frac{\psi}{\psi + (1-\sigma)\left(\dfrac{(\tilde{\pi} + w_s h)^+}{1 - (\tilde{\pi} + w_s h)^+} \right)} \right|$$ Supp. Eq. (2b)

$$M_-(h) = \left| \mathrm{NPV_e} - \frac{\psi}{\psi + (1-\sigma)\left(\dfrac{(\tilde{\pi} - w_s h)^+}{1 - (\tilde{\pi} - w_s h)^+} \right)} \right|$$ Supp. Eq. (2c)

We are again using the function x^+ defined earlier.
